# Priority medicines for Europe and the World: setting a public-health-based medicines development agenda

**DOI:** 10.1186/2052-3211-8-S1-K4

**Published:** 2015-10-05

**Authors:** Veronika J Wirtz

**Affiliations:** 1 Department of Global Health, Boston University School of Public Health, Boston, MA 02118, USA

## Background

This presentation will summarize key results of the 2013 report Priority Medicines for Europe and the World [1] which is an update to the original 2004 report and takes into account changes in global health and pharmaceutical innovation since 2004.

## Methods

Using a global public health perspective for Europe and the world and based on the principle of equity and efficiency the report identifies gaps in the development and research of pharmaceutical technologies which are needed to meet the priority health care needs of the population but which have not yet been developed. Four criteria were used to determine the gaps: the burden of disease in Europe, its trend, common risk factors amenable to pharmacological intervention and the principle of "social solidarity". The gaps are divided into three groups depending whether treatment exist, how effective it is and whether the delivery mechanisms or formulation are appropriate of the target population.

## Results

(1) Antibacterial resistance and pandemic flu are two areas where treatments exist but may become ineffective soon.

(2) Treatment options are available for cardiovascular diseases, HIV, cancer, depression, diabetes, pneumonia, diarrhoea, neonatal diseases, malaria, tuberculosis, neglected tropical diseases and postpartum haemorrhage but the pharmaceutical delivery mechanisms or formulations are not appropriate for the target populations.

(3) For stroke, osteoarthritis, Alzheimer's disease and other dementias, chronic obstructive pulmonary disease, hearing loss and low back pain, treatment does not exist or is not sufficiently effective.

## Discussion

To provide incentives for development to fill these pharmaceutical technology gaps innovations are needed in the areas of market regulation as well as pricing and reimbursement policies. To respond to new knowledge that is being produced about the pharmaceutical technologies multiple market authorization and reimbursement decisions over time may be required instead of a single decision at one point in time. For example, there seem to be large room for improvement of how to use electronic medical records. To further develop value-based pricing and adaptive licensing new methods for evidence generation, benefit risk assessment and regulatory dialogue need to be developed.

## References

[B1] Kaplan W Wirtz VJ Mantel-Teeuwisse A Stolk P Duthey B Laing R Priority Medicines for Europe and the World 2013 Update 2013 Geneva: World Health Organization in collaboration with Utrecht University and Boston University

